# FRLTC: A new topology control algorithm using federated reinforcement learning for software-defined wireless sensor network in IoT

**DOI:** 10.1371/journal.pone.0355604

**Published:** 2026-08-17

**Authors:** Le Huu Binh, Thuy-Van T. Duong, Duc Huy Le

**Affiliations:** 1 Faculty of Information Technology, University of Sciences, Hue University, Hue City, Vietnam; 2 Faculty of Information Technology, Ton Duc Thang University, Ho Chi Minh City, Vietnam; 3 Faculty of Information Technology, Ha Noi University of Business and Technology, Hanoi, Vietnam; Maha Bharathi Engineering College, INDIA

## Abstract

Topology is one of the most important factors influencing the performance of software-defined wireless sensor networks (SDWSN) in the Internet of Things (IoT). This paper presents a novel topology control algorithm using federated reinforcement learning, proposed for SDWSN in the IoT. Our approach represents the SDWSN as a federated learning system in which each sensor node runs a reinforcement learning model to change its communication range such that the node degree approaches the desired value. Another reinforcement learning model was applied to the SDN controller to assign the sensor node for learning, bringing the average degree of the entire network closer to the desired value. Simulation results reveal that the proposed approach outperforms well-known topology control algorithms in terms of the desired node degree, energy consumption, and quality of transmission.

## Introduction

**Fig 1:**
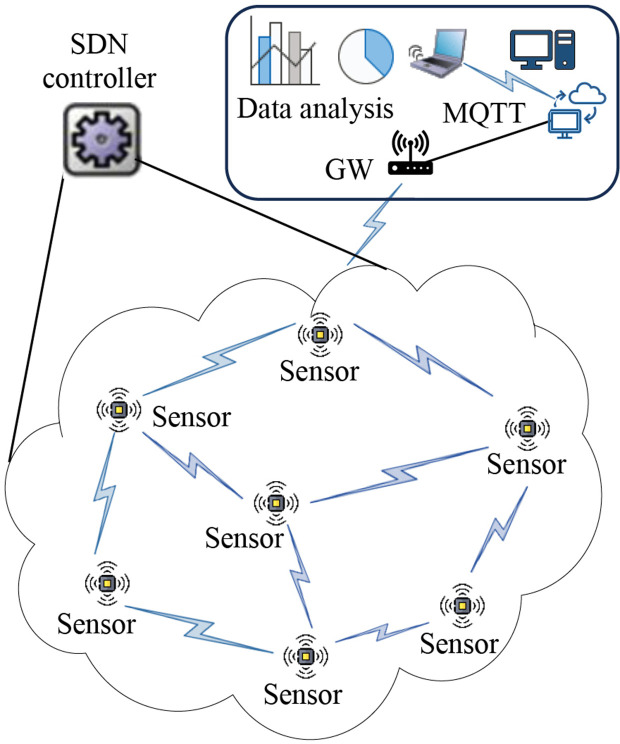
An example of the SDWSN in IoT.

The Internet of Things (IoT) is becoming increasingly important in modern life. A wireless sensor network (WSN) is an important component for collecting and transmitting information from sensor devices to an IoT control center. In the context of increasingly explosive IoT applications, the technologies used in WSN must be changed to better satisfy the task of transmitting information between sensor devices. Software-defined WSN (SDWSN) are a recent trend in WSN technology that has been researched and deployed recently [1,2]. Fig 1 shows an example of an SDWSN in IoT, where sensor nodes are connected directly or indirectly (through other nodes) to the SDN controller using an open flow protocol. The SDN controller centrally performs control functions in the network, such as routing, signaling, and topology control. The sensor nodes in a WSN transmit the collected data to the base station (BS). The BS data were transmitted to the server system via the internet. The message queuing telemetry transport (MQTT) protocol is often used to communicate between sensor nodes, server systems, and their users.

**Fig 2:**
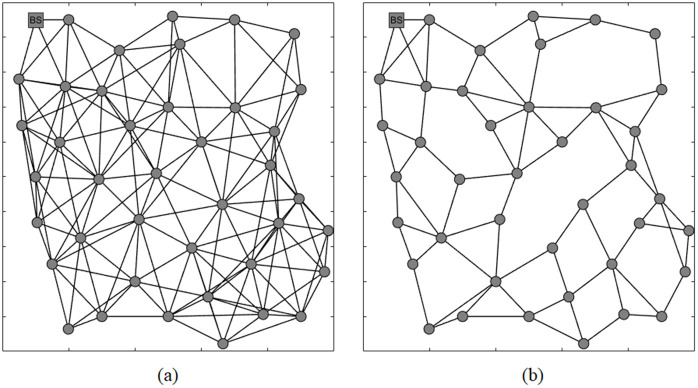
Comparison of WSN topologies in different cases. (a) no topology control, (b) topology control with desired node degree of 3.

To ensure the quality of service (QoS) of IoT applications, it is necessary to improve the performance of wireless sensor networks (WSN. The network topology significantly influences the performance of WSN. According to the general principle of wireless ad hoc networks, a wireless link is created between nodes when they are within the coverage area of each other. Therefore, there are cases in which the network topology contains many wireless links, including unused links, particularly for WSN that use many sensor nodes and have a high node density. Fig 2 shows an example of the network topology for cases with and without topology control. The topology in Fig 2(a) corresponds to the case without topology control, in which the sensor nodes operate at their maximum transmission power. This is the default case for wireless sensor networks (WSNs). The advantage of this topology is that multiple paths exist between the sensor nodes and between each sensor node and the base station. However, this method has several limitations. However, it has several disadvantages, the most serious of which is that nodes must maintain multiple wireless connections, resulting in high energy consumption. In addition, the presence of multiple wireless connections leads to nodes frequently receiving many control packets from their neighbors, such as hello and beacon packets. This occupies a large bandwidth of the transmission channels, which can lead to network congestion owing to the control packets. The topology shown in Fig 2(b) corresponds to the case in which a topology control algorithm was used. The number of wireless connections between the sensor nodes was maintained at a moderate level based on the desired node degree,. This topology minimizes the energy consumption at each sensor node, which is suitable for WSN systems in the IoT, where there is no need for an excessively large traffic load.

The network topology must contain only the necessary connections, as shown in Fig 2(b), the topology control problem must be considered, particularly for WSNs with high node density. This problem in WSN is stated as follows: Given a network area and a set of nodes, each node is a sensor with given technical parameters. Topology control involves adjusting technical parameters, the location of sensor nodes, or other factors to ensure that the resulting network topology is optimal according to the set goals. The most basic objective often considered in the topology control problem is to obtain the average degree of a node closer to a desired value to optimize the use of energy [3,4], reduce the signal power loss [5], and increase the coverage area of the entire network [6]. In a network topology, the degree of a node is determined by the number of neighboring nodes, that is, the number of wireless connections to a node.

Topology control in a WSN is an NP-hard problem, which means that it is impossible to find an exact solution with the complexity of a polynomial function. Approximation methods are often used to solve these problems. Recently been used include graph theory [7,8], optimization algorithms [4,9], and machine learning [3,10]. Among these methods, topology control using machine learning is the most suitable for wireless ad-hoc network systems. The application of machine learning techniques suitable for topology control algorithms requires high-performance computing and rapid adaptation to network systems with frequent state changes. Thien et al. [3] proposed an energy-efficient topology control addedmethodfor WSN, called RL-CRC, based on reinforcement learning (RL). Their solution used an RL algorithm to change the communication range of each node to achieve an optimal architectural design. Using simulation data, the authors demonstrated that the RL-CRC algorithm consumes less energy at the nodes than conventional topology control algorithms. The authors of [11] suggested a topology optimization approach for self-organized energy-efficient WSNs based on deep reinforcement learning. To launch the simulations, the authors employed a deep neural network to direct a Monte Carlo tree search, and the outcomes of the tree search supported the learning of the neural network. Numerous simulations demonstrated that the proposed approach outperformed heuristic solutions and could be adjusted to changes in the network and environment without restarting the procedure. In [12], a topology control algorithm based on fuzzy logic was applied. To increase network connectivity, the authors of this paper suggest a novel topology control algorithm for WSN called fuzzy logic-based topology control (FTC). To accomplish this, the FTC algorithm modifies each node’s communication area so that the average node degree approaches the desired degree. Using simulation approaches, the performance of the FTC algorithm was compared with that of well-known topology-control algorithms. In [10], the authors suggested a reinforcement learning-based topology control system for drone networks that first determines the relative positions of UAVs in a swarm, then optimizes the connectivity between them in terms of interference and energy consumption, and finally reshapes the logical structure of drone networks by selecting neighbors for each UAV and mapping the data flows over them. The performance of the proposed system was confirmed through theoretical research and simulation tests on several UAV topologies.

Based on the research studies reviewed above, we conclude that applying machine learning to the topology control problem results in high efficiency. However, several problems remain to be addressed, particularly in next-generation wireless network systems. This was the motivation for this study. We propose a new and effective method for applying machine learning to the topology control problem in SDWSN in the IoT. The main contributions of this study are as follows:

(i) We propose a federated reinforcement learning (FRL) model for the topology control problem in SDWSN, in which reinforcement learning is applied at each sensor node to appropriately adjust its transmission power. Another reinforcement learning model is applied to the SDN controller to assign sensor nodes to perform learning, such that the average node degree in the entire network approaches the desired degree. To the best of our knowledge, this is the first study to apply federated learning to the topology control problem in an SDWSN.
(ii) We propose a new topology control algorithm for WSNs called Federated Reinforcement Learning-based Topology Control (FRLTC). The simulation results showed that the FRLTC algorithm outperformed the state-of-the-art topology control algorithms in terms of the desired node degree, energy consumption, and quality of transmission.

The remainder of this paper is organized as follows. The next section presents the FRL model for the topology control problem in the SDWSN and the proposed algorithm. The experimental results are presented in this section. The final section presents the conclusions and suggestions for future research.

## FRL-based topology control for SDWSN in IoT

### FRL model for topology control problem

Reinforcement learning (RL) [13] is a subfield of machine learning in which an agent learns to make decisions through constant interactions with its environment. In reinforcement learning, the purpose of the agent is to optimize its behavior to achieve the maximum overall reward value during the learning process. An RL model is typically defined by five components: the environment, agent, state, action, policy, and reward.

**Fig 3:**
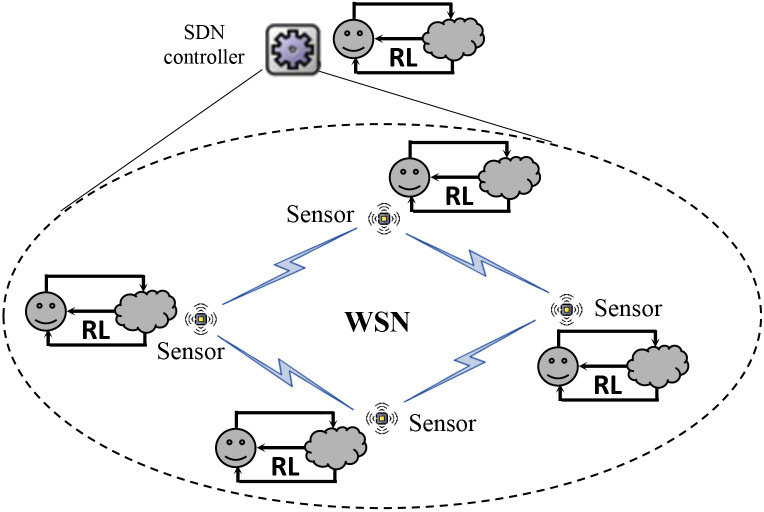
Federated reinforcement learning for topology control in SDWSN.

When applied to wireless networks, the RL is typically distributed at each network node [3,6]. For the topology control problem, implementing only the distribution at each node is inefficient because the node states are interdependent. To improve the efficiency of applying reinforcement learning to topology control in SDWSN, we propose an FRL model, as shown in Fig 3. The RL is applied at each sensor node to adjust the transmit power flexibly to bring the node degree closer to the desired value. Another RL model is applied to the SDN controller to select the sensor node to perform learning with the goal that the average degree of all nodes quickly converges to a desired value. The RL model at the SDN controller uses data from the learning results of the RL models at the sensor nodes to decide which sensor nodes perform the learning so that the algorithm converges quickly.

For the FRL model of the topology control problem, as shown in Fig 3, the five components of RL, namely, environment, agent, state, action, policy, and reward at the sensor nodes and SDN controller are modeled differently, as detailed in the subsections below.

### RL model at each sensor node

**Fig 4:**
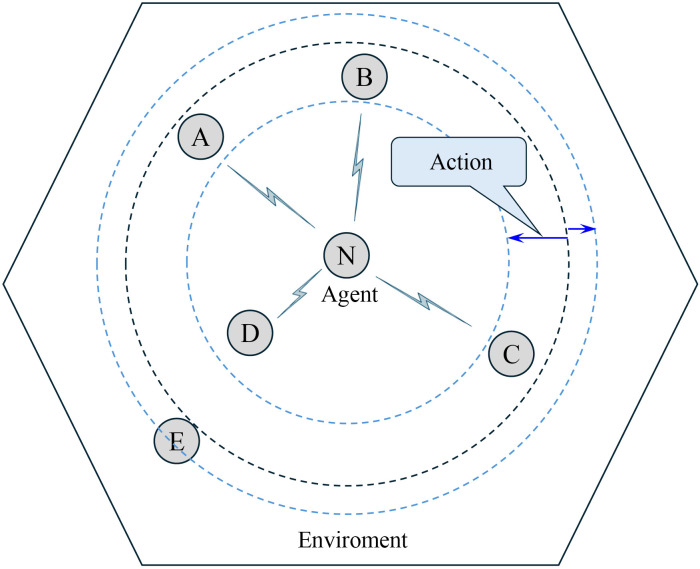
RL model at each sensor node.

The RL at the sensor nodes learns to adjust the communication range by changing its transmission power to obtain the desired node degree, as follows: Fig 4 shows the application of RL at the sensor nodes.

**Environment.** The environment exists around the agent, where the agent operates and interacts. For the topology control problem in an SDWSN, the environment is the network system, which is a set of all nodes, wireless connections, and factors that affect the performance of the network system, such as thermal noise and frequency noise from devices outside the network system.

**Agent.** For the topology control problem in an SDWSN, the agents can be different components, depending on the implementation method. In our context, topology control is performed by adjusting the communication range of each agent, which is a node that must regularly adjust the transmit power or receive sensitivity to change its communication range.

**State.** The state is perceived by the agent. In this model, each state is determined by the degree of a node, that is, the number of neighbors of that node. In an SDWSN with *n* sensor nodes, the possible states are distributed in [1,n−1]. Let $S_{sn}$ be the set of states, then $S_{sn}$ is given by


$$\begin{array}{c} S_{sn}=\{s|s=\overset{―}{1..n-1}\} \end{array}$$ (1)

**Action.** The action by which an agent interacts with the environment is to adjust its communication domain such that the degree of the node approaches its desired value. Let $A_{sn}$ be the action space that the agent (i.e., sensor node) can choose to perform, then $A_{sn}$ is determined by


$$\begin{array}{c} A_{sn}=\{r_{min},\;r_{min}+\beta ,\;r_{min}+2\beta ,...,\;r_{max}\} \end{array}$$ (2)

where β represents the step between actions and $r_{min}$ and $r_{max}$ represent the minimum and maximum communication ranges, respectively.

**Reward.** In topology control problems, the reward function is used as the objective. A common objective of these problems is that the degrees of the nodes approach a given desired degree value [3,5,7]. In this work, the reward function is designed as follows


$$\begin{array}{c} R_{sn}(i,s,a)=1-\frac{\left|k_{local\_current}-k_{desired}\right|}{k_{max}-k_{desired}} \end{array}$$ (3)

where $k_{local\_current}$ represents the current degree of node *i*, $k_{max}$ represents the maximum degree of all nodes in the network, and $k_{desired}$ represents the desired degree of nodes. In RL, the total reward when an agent performs an action is determined by the Q-learning algorithm [14], which is updated according to the Bellman equation as follows:


$$\begin{array}{c} Q_{sn}(i,s,a)=(1-\alpha )Q_{sn}(i,s,a)+\alpha [R_{sn}(i,s,a)+\gamma \underset{\forall a^{\prime }\in A}{max}Q_{sn}(i,s^{\prime },a^{\prime })] \end{array}$$ (4)

where α and γ∈[0,1] are the learning rate and discount factors, respectively; $s^{\prime }$ denotes the next state reached after executing action a in state *s*, and $a^{\prime }$ represents a candidate action available at state $s^{\prime }$ used in the maximization operator to estimate the optimal future Q-value.

**Policy.** In RL, action selection is performed based on policies. One policy that has been used for topology control problems based on RL is ε-*greedy* [3,6]. For this policy, the action that yields the largest reward value is selected with a high probability of 1−ε. The remaining actions are selected with a low probability, equal to ε.

### RL model at SDN controller

To implement RL in the SDN controller, the operation of the SDN controller must be modeled as an RL model. The environment and policy components were similar to those of the RL model at the sensor node. The agent corresponds to the SDN controller, which learns to select a sensor node to perform a communication range adjustment. The state is determined by the average degree of all nodes in the network. Let $S_{sdn}$ be the state space of RL at SDN controller, then $S_{sdn}$ is determined by


$$\begin{array}{c} S_{sdn}=\{d_{avg}^{\left(min\right)},d_{avg}^{\left(min\right)}+\delta ,d_{avg}^{\left(min\right)}+2\delta ,...,d_{avg}^{\left(max\right)}\} \end{array}$$ (5)

where δ represents the step of the states, $d_{avg}^{\left(min\right)}$ and $d_{avg}^{\left(max\right)}$ are the minimum and maximum average degrees of all nodes when the transmit powers of the nodes are minimum and maximum, respectively. Each agent action corresponds to assigning a sensor node to learn and adjust the communication range. Let $S_{sdn}$ be the set of feasible actions that an agent can perform in the RL model at SDN controller, then $A_{sdn}$ is given by


$$\begin{array}{c} A_{sdn}=\{a_{1}^{\left(sdn\right)},a_{2}^{\left(sdn\right)},a_{3}^{\left(sdn\right)},...,a_{n}^{\left(sdn\right)}\} \end{array}$$ (6)

where $a_{i}^{\left(sdn\right)}$ represents the action that *i* node is selected to adjust its communication range. The goal of the RL model at the SDN controller is to obtain the average node degree in the entire network that is closest to the desired value. Therefore, the reward is designed as follows


$$\begin{array}{c} R_{sdn}(i,s,a)=1-\frac{\left|{\overset{―}{k}}_{global\_current}-k_{desired}\right|}{{\overset{―}{k}}_{max}-k_{desired}} \end{array}$$ (7)

where ${\overset{―}{k}}_{global\_current}$ represents the current average degree, ${\overset{―}{k}}_{max}$ represents the maximum average degree, and $k_{desired}$ represents the desired degree of nodes. The total reward when an agent performs an action is determined by the Q-learning algorithm, determined by


$$\begin{array}{c} Q_{sdn}(i,s,a)=(1-\alpha )Q_{sdn}(i,s,a)+\alpha [R_{sdn}(i,s,a)+\gamma \underset{\forall a^{\prime }\in A_{sdn}}{max}Q_{sdn}(i,s^{\prime },a^{\prime })] \end{array}$$ (8)

where α and γ∈[0,1] are the learning rate and discount factor, respectively.

### FRLTC algorithm

#### Federated decision-level interaction mechanism.

**Fig 5:**
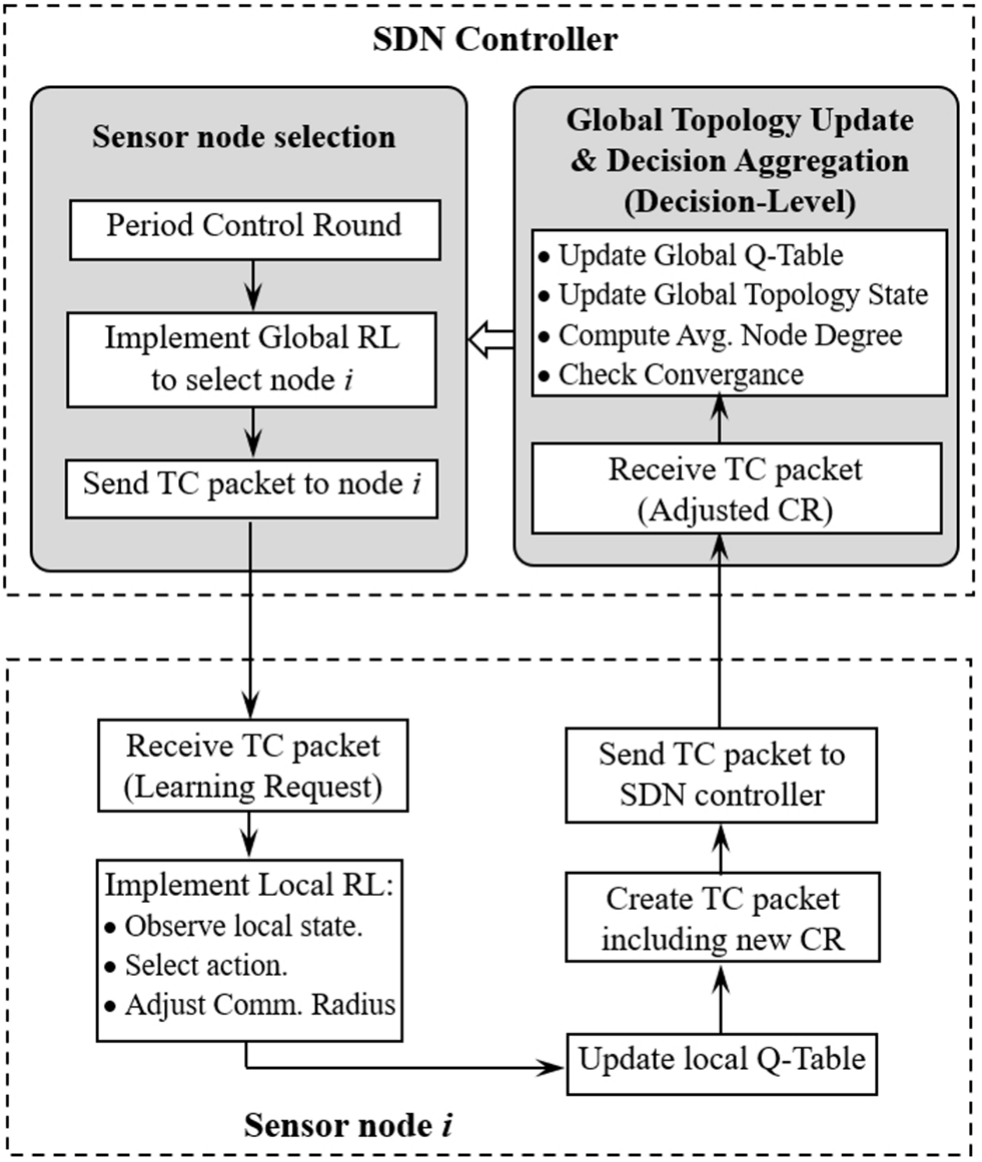
Overall federated decision-level interaction workflow between the SDN controller and sensor nodes in the proposed FRLTC algorithm.

Fig 5 illustrates the federated decision-level reinforcement learning workflow of the proposed FRLTC framework. The learning process is organized into periodic control rounds that are initiated by the SDN controller. At the beginning of each round, the controller applies a global reinforcement learning policy to select a sensor node for adjusting the communication range. A topology control (TC) packet containing the learning request is then transmitted to the selected nodes.

Upon receiving the TC packet, the sensor node performs local reinforcement learning based on its observed state, selects an action, and adjusts its communication radius. The local Q-table was updated using the rewards obtained. The node then creates a TC packet containing the adjusted communication radius (CR) and sends it back to the SDN controller.

After receiving the updated CR, the controller performs a decision-level aggregation by updating the global Q-table and reconstructing the global topology state. The current average node degree was computed, and a convergence check was conducted. If the desired topology condition is not achieved, the controller initiates the next round of control. Notably, aggregation occurs at the decision level rather than at the model parameter level. No raw data or neural network parameters were exchanged between nodes and the controller. Instead, only the adjusted communication radius is reported, which significantly reduces the communication overhead while preserving decentralized learning and centralized coordination.

**Algorithm 1 Select sensor nodes to adjust communication range using reinforcement learning at the SDN controller**

*// Initialization*

1:  Implement the minimum spanning tree algorithm to determine the minimum communication range, $r_{min}$, for all sensor nodes;

2:  Compute the average node degrees, $d_{avg}^{\left(max\right)}$ and $d_{avg}^{\left(min\right)}$, for the cases where the communication ranges of the nodes are $r_{max}$ and $r_{min}$, respectively;

3:  Determine the sets of states and actions, $S_{sdn}$ and $A_{sdn}$, using (5) and (6), respactively;

4:  $Q_{sdn}(s_{i}^{\left(sdn\right)},a_{i}^{\left(sdn\right)})=0\;\forall s_{i}^{\left(sdn\right)}\in S_{sdn},\forall a_{i}^{\left(sdn\right)}\in A_{sdn}$;

*// Learning process*

5:  **while** (**true**) **do**

6:   wait(*interval*);

7:   Select action $a_{i}^{\left(sdn\right)}\in A_{sdn}$ according to ε-greedy policy;

8:   Send TC packet to node *i*;

9:   Waiting for TC packet from node *i*;

10:   **if** (*TC packet is received* **and** $T_{w}<=T_{limit}$) **then**

11:    Read the adjusted communication range of node *i* stored in the TC packet;

12:    Update $Q_{sdn}(s_{i}^{\left(sdn\right)},a_{i}^{\left(sdn\right)})$ using (8);

13:   **end if**

14:  **end while**

#### Learning procedure for communication range adjustment.

The learning process at the SDN controller to select the sensor node to perform communication range adjustment is performed according to Algorithm 1. In the initialization phase, the SDN controller first executes the minimum spanning tree algorithm to determine the minimum communication range, $r_{min}$, for all sensor nodes (Step 1). The sensor nodes only adjust their communication area within the range $\left[r_{min},r_{max}\right]$ to ensure connectivity in the network. In the learning stage, periodically after each *interval*, the agent performs learning, selects a sensor node according to policy ε-*greedy* (Step 7), saves the information into a topology control (TC) packet, and sends it to the selected sensor node (Step 8). The agent then waits to receive a response from the sensor node (Step 9). If the agent receives a response packet within the allowed timeout period, it updates the Q-value according to (8) (Step 12). When a sensor node receives a TC packet from the SDN controller, it learns to adjust its communication range accordingly. This task is performed according to Algorithm 2, and the learning result is placed into a TC packet to be sent to the SDN controller for processing.

**Algorithm 2 Adjust the communication range at each node using reinforcement learning**

*// Initialization*

1:  Determine the sets of states and actions, $S_{sn}$ and $A_{sn}$, using (1) and (2);

2:  $Q_{sn}(s_{i}^{\left(sn\right)},a_{i}^{\left(sn\right)})=0\;\forall s_{i}^{\left(sn\right)}\in S_{sn},\forall a_{i}^{\left(sn\right)}\in A_{sn}$;

*// Learning process*

3:  **if** (*TC packet is received from SDN controller*) **then**

4:   Select action $a_{i}^{\left(sn\right)}\in A_{sn}$ according to ε-greedy policy;

5:   Adjust the communication range based on the selected $a_{i}^{\left(sn\right)}$ action;

6:   Update $Q_{sn}(s_{i}^{\left(sn\right)},a_{i}^{\left(sn\right)})$ using (4);

7:   Create TC packet, store adjusted communication range into it;

8:   Send TC packet to SDN Controller;

9:  **end if**

#### TC packet overhead.

In the proposed algorithm, the learning interaction between the SDN controller and sensor nodes relies solely on lightweight TC packets rather than full model parameters or gradient exchanges, as in conventional federated learning. Each update involves only a single request–response exchange with one selected node per control interval, and the transmitted information is limited to compact state/action indicators and the adjusted communication range value. Because the control interval is significantly larger than the data transmission interval, the resulting communication overhead remains small compared to regular traffic. Furthermore, by optimizing the communication range and reducing redundant links, the proposed scheme decreases the overall network transmission, thereby compensating for the minor energy cost introduced by the TC packet exchanges.

## Performance evaluation

### Simulation scenarios

To evaluate the performance of the proposed algorithm, FRLTC, we set up simulations using OMNeT++ [15] and the INET framework [16]. The FRLTC algorithm was compared with the RTLR [7], TFACR [5], and ROATC [17] algorithms and the case of without topology control, called MaxPower. The metrics examined included the node degree, energy expansion ratio and path loss. MaxPower is the default case in ad hoc wireless networks and SDWSN. Sensor nodes always operate with the maximum communication range, according to wireless communication standards.

Table 1 presents the technical parameters set for the simulation scenarios. The SDWSN was deployed in an area of $1000\times 1000[m^{2}]$. The number of sensor nodes ranged from 40 to 80, depending on the simulation scenario, and their coordinates were randomly distributed in the network. The maximum communication range of the sensor nodes was 250 [*m*].

**Table 1:** The parameters of simulation scenarios.

Parameter	Setting
The area of SDWSN	$1000\times 1000\;[m^{2}]$
Number of sensor nodes	From 40 to 80
Maximum communication range	250 [*m*]
Desired node degree	From 3 to 5
Signal power loss model	Free space
Topology control algorithms	MaxPower, LTRT, TFACR, ROATC and FRLTC
Learning rate factor (α)	0.7
Discount factor (γ)	0.3
ε factor in ε-*greedy* policy	0.1

### Simulation results

#### Analyzing node degree.

**Fig 6:**
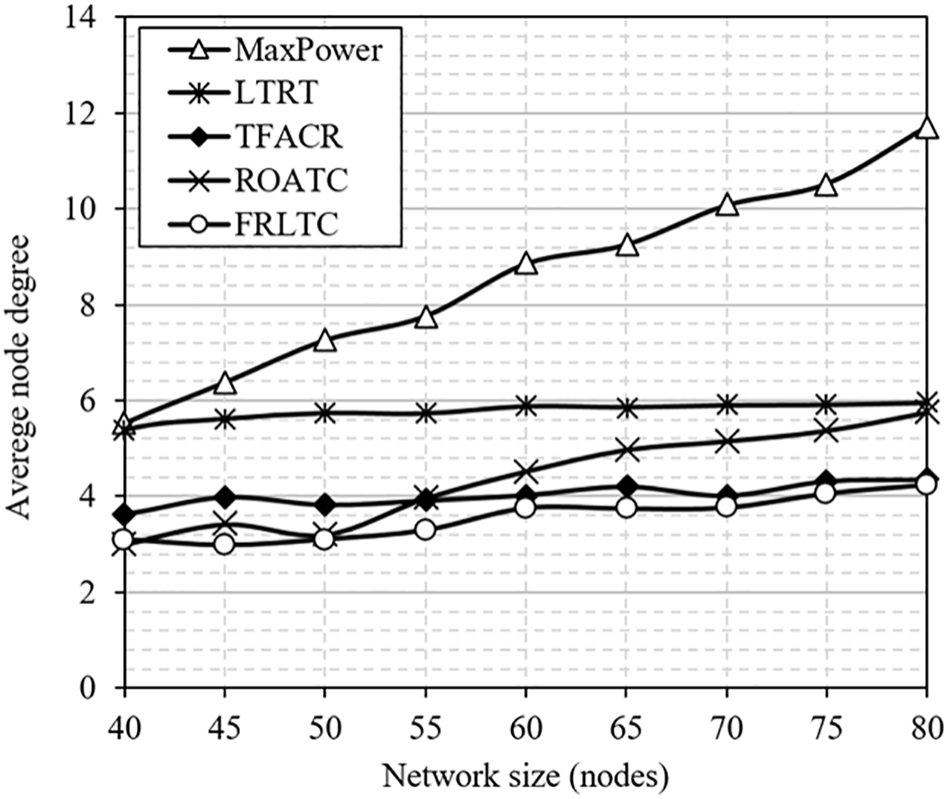
Average node degree versus network size in case of desired degree of 3.

First, we analyzed the node degree, which is a commonly used metric for evaluating the optimality of network topology. The results in Fig 6 show the average node degree versus network size in case of desired degree of 3. We can observe that, in the case of MaxPower, the difference between the average node degree and the desired degree is very large, especially when the SDWSN uses many sensor nodes. This is because, in this case, the sensor nodes operate with the maximum communication range; therefore, in the same network area, the more the number of sensor nodes, the more the sensor nodes are in the coverage area of the other nodes. A network topology with a high average degree, such as MaxPower, has many disadvantages, especially when applied to the IoT, where the traffic load is usually not large. The most serious drawback is that nodes must maintain multiple wireless connections, resulting in high-energy consumption. In addition, the presence of multiple wireless connections leads to nodes frequently receiving several control packets from their neighbors. In cases where topology control algorithms were used, the average node degree approached the desired value. Comparing the four topology control algorithms, LTRT, TFACR, ROATC, and FRLTC, we can easily observe that the proposed FRLTC provides an average degree closest to the desired degree. Considering the scenario of 50 sensor nodes, the average degrees of the LTRT, TFACR, ROATC, and FRLTC algorithms were 5.74, 3.80, 3.20, and 3.10, respectively. Compared with the desired degree of this scenario of 3, the FRLTC algorithm differed by only 0.1. Although the ROATC also achieved an average degree relatively close to the desired value, its deviation (0.2) was still higher than that of the FRLTC. For other cases of the number of sensor nodes, FRLTC consistently outperformed ROATC and the other benchmark algorithms by maintaining an average node degree that was closest to the desired value. Consequently, the FRLTC algorithm always provides an average degree that is closest to the desired degree.

**Fig 7:**
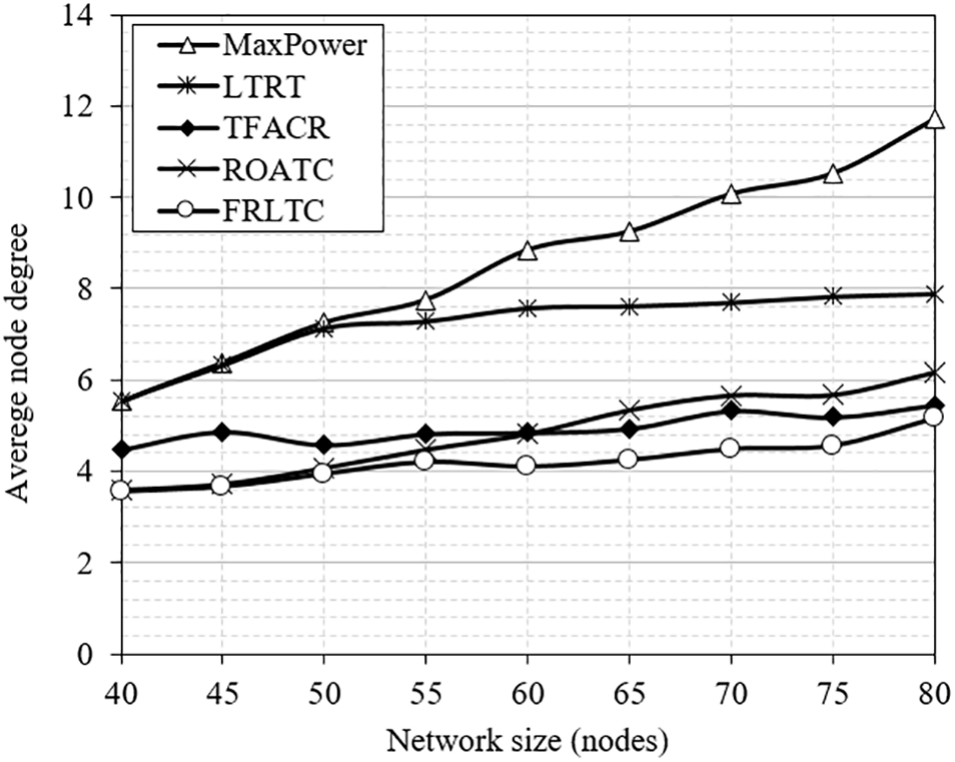
Average node degree versus network size in case of desired degree of 4.

The results are identical for the case in which the desired degree is four, as shown in Fig 7. For all simulation scenarios in which the number of sensor nodes ranged from 40 to 80, the average degrees of the LTRT, TFACR, ROATC, and FRLTC algorithms were 5.53 to 7.98, 4.47 to 5.46, 3.60 to 6.11 and 3.55 to 5.11, respectively. Thus, the FRLTC algorithm provides a more optimal average node degree than the other two algorithms.

**Fig 8:**
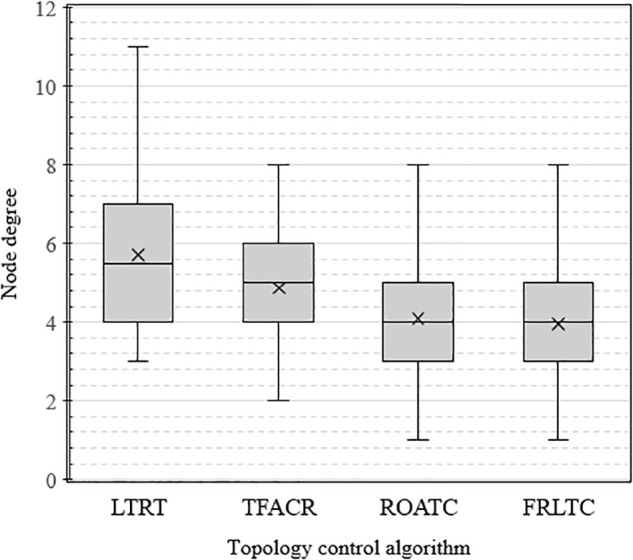
Node degree distribution of LTRT, TFACR, ROATC and FRLTC algorithms in case of number of sensor nodes of 50 and desired degree of 4.

To clearly observe the specific degree values of all nodes in the network, we present the details using box plots, as shown in Fig 8. In the case of the LTRT algorithm, the node degrees range from 3 to 11, with median and mean values of 5.5 and 5.75, respectively, of which 20% are in the range of 7–11. This result shows that many nodes exist whose degrees are far from the desired degree. In the case of the TFACR algorithm, the degrees of the nodes converged better to the expected values in the region from 2 to 8, with median values of 5.0 and 4.6, respectively. Similarly, the ROATC algorithm produces node degrees ranging from 1 to 8, with median and mean values of 4.0 and 4.1, respectively, indicating a better node degree distribution than that of TFACR. The FRLTC algorithm case yielded the best node degree distribution, ranging from 1 to 8, with median and mean values of 4.0 and 3.94, respectively. Compared with ROATC, FRLTC achieves a slightly lower mean node degree while maintaining the same median, demonstrating that its node degrees are distributed closer to the desired values. This result clearly shows the efficiency of the FRLTC algorithm in terms of node degree, compared with the other three algorithms.

#### Analyzing Energy Expansion Ratio (EER).

**Fig 9:**
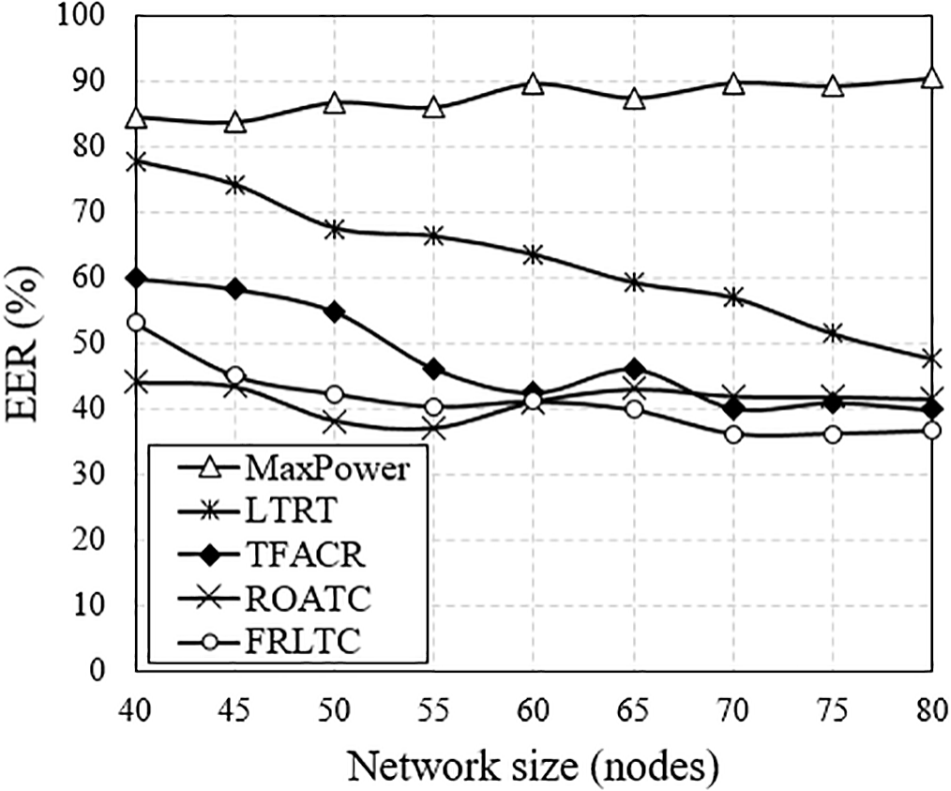
EER versus network size in case of desired degree of 3.

Next, we analyzed the EER, which is an important metric for the SDWSN. Next, we analyzed the energy expenditure ratio (EER) of the network. In this study, the EER was calculated similarly to that in [3,18]. The results in Fig 9 show EER versus network size in the case of desired degree of 3. We can observe that, in the case of MaxPower, the EER is very large, ranging from 84.45 to 90.41%. This leads to the rapid exhaustion of the energy resources of the sensor nodes. In the case of topology control with the LTRT, TFACR, ROATC, and FRLTC algorithms, the EER was significantly decreased. The EER of these algorithms was in the range of 47.67 to 77.66%, 39.83 to 59.91%, 37.81 to 43.86%, and 36.70 to 53.11%, respectively. Thus, the FRLTC algorithm yielded the lowest EER value. When comparing the four topology control algorithms, it can be observed that ROATC achieves the lowest EER for network sizes below 60 sensor nodes. However, as the network size exceeds 60 sensor nodes, the proposed FRLTC algorithm outperforms ROATC and consistently achieves the lowest EER. This demonstrates that FRLTC is more energy-efficient and scales better in dense SDWSN deployment.

**Fig 10:**
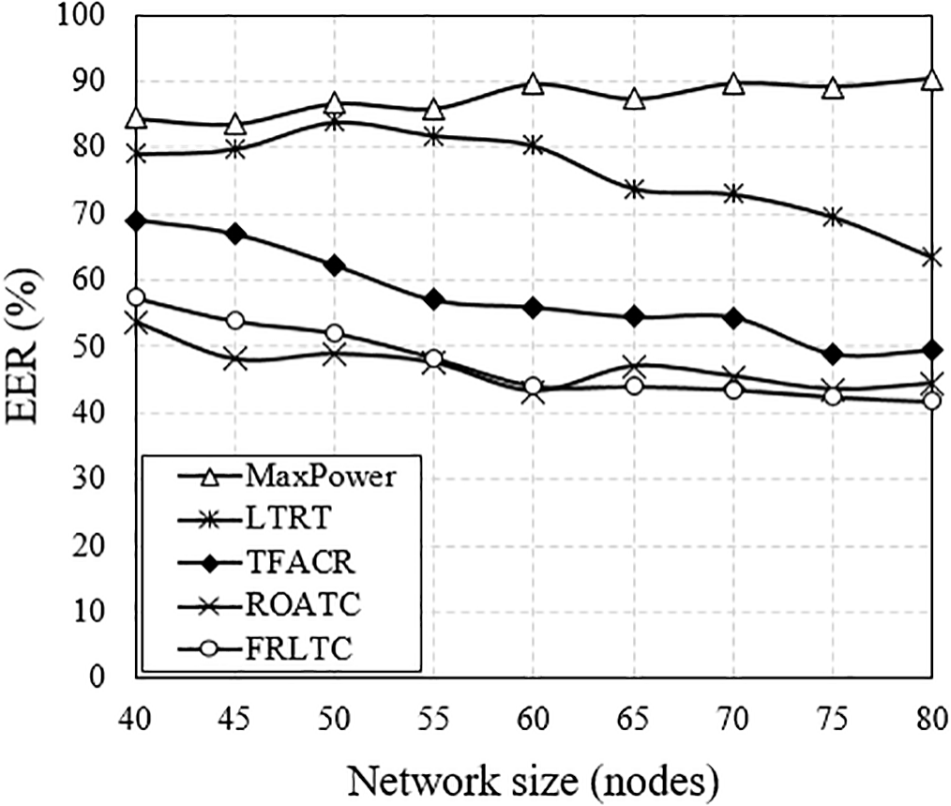
EER versus network size in case of desired degree of 4.

In the simulation scenario in which the desired degree was 4, the EER of all algorithms increased owing to the existence of more wireless connections and wider communication range of the nodes. This is illustrated in Fig 10. However, among these algorithms, the FRLTC yielded the lowest EER. These results demonstrate that the FRLTC algorithm outperforms the other algorithms in terms of energy efficiency. This allows the lifetime of the sensor nodes to be extended, thereby improving the network performance.

#### Analyzing path loss.

**Fig 11:**
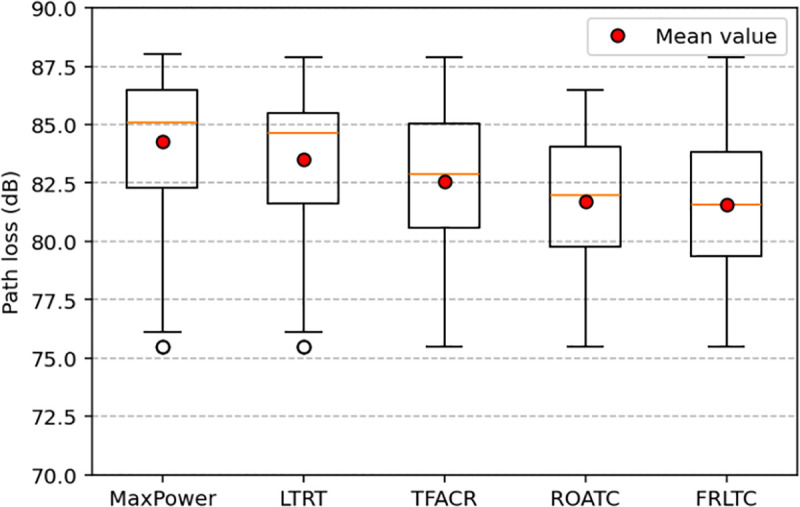
Comparison of path loss of MaxPower, LTRT, TFACR, ROATC and FRLTC algorithms in case of desired degree of 3.

**Fig 12:**
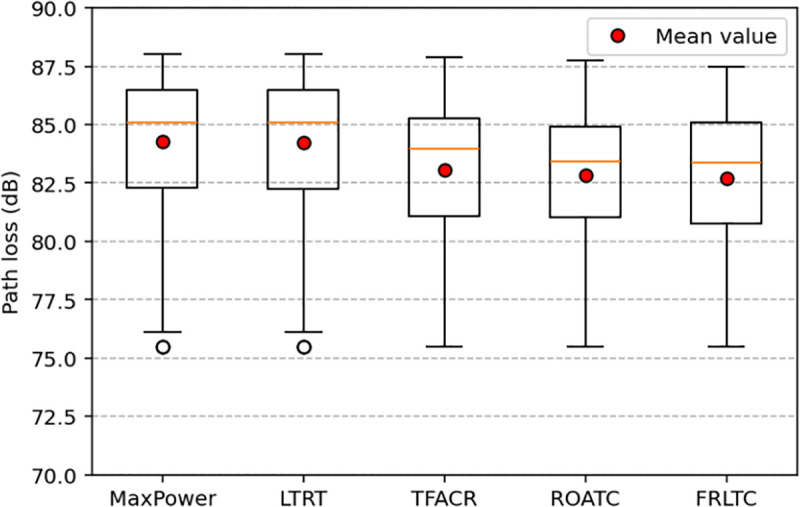
Comparison of path loss of MaxPower, LTRT, TFACR, ROATC and FRLTC algorithms in case of desired degree of 4.

**Fig 13:**
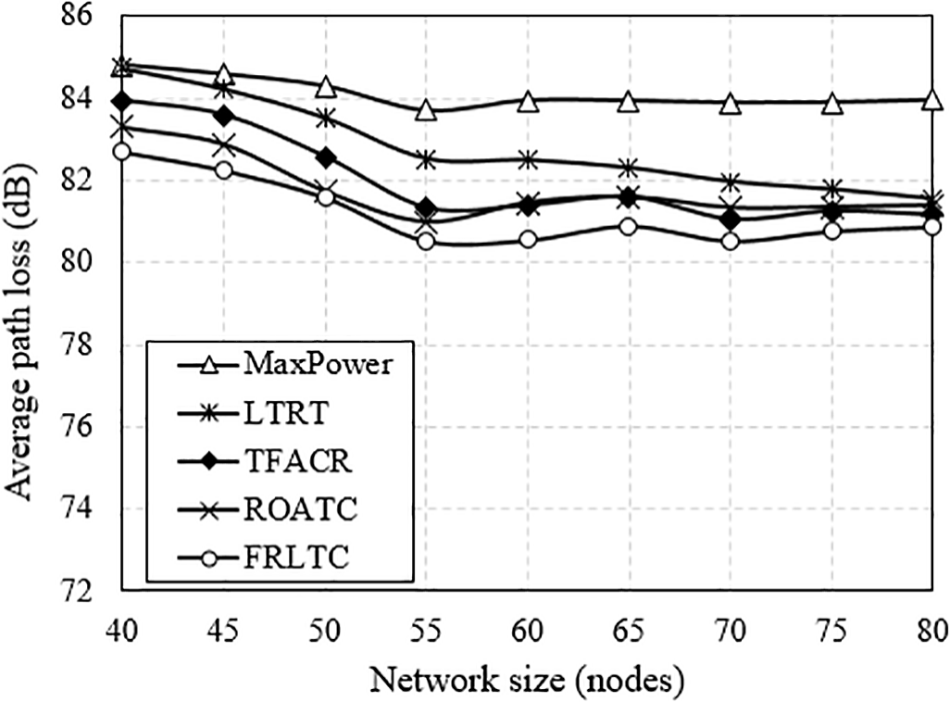
Path loss versus network size in case of desired degree of 3.

**Fig 14:**
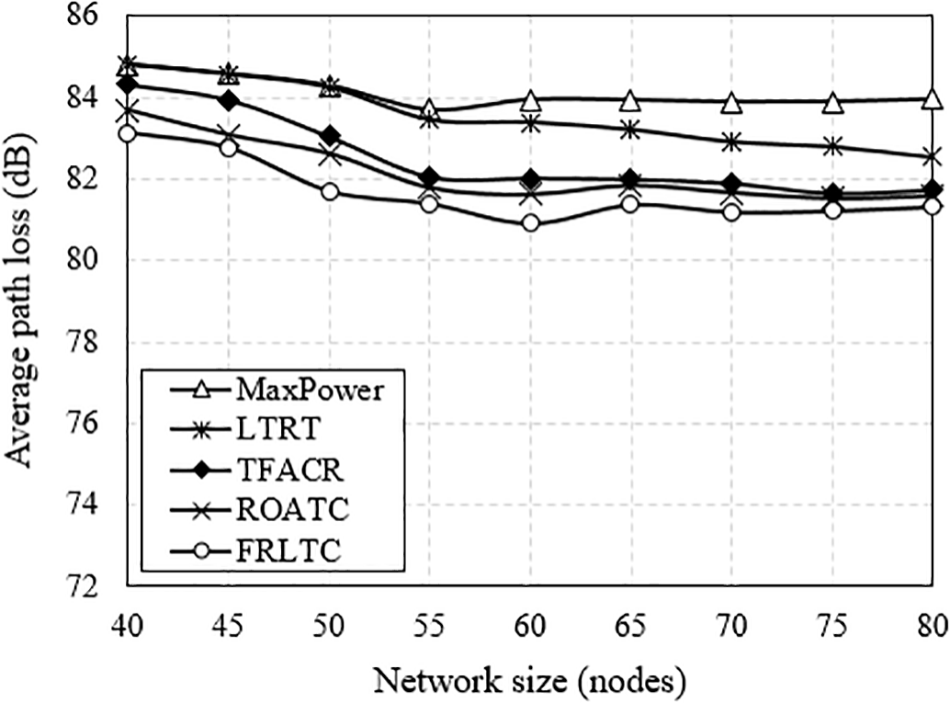
Path loss versus network size in case of desired degree of 4.

In a wireless communication environment, there is always a path loss between the source and destination nodes. Path loss significantly influences the transmission quality in a network. We investigated this metric, and the results are shown in Fig 11 to 14. The results in Figs 11 and 12 are executed on a simulation scenario with the number of sensor nodes of 50 and desired degrees of 3 and 4, respactively. We can observe that the path loss range of all the algorithms is similar, ranging from 75.5–80.0 dB. However, the distribution density is different, as shown by the mean and median values in the box charts, which are summarized in Table 2. Compared with TFACR, the ROATC algorithm slightly reduced both the mean and median path loss values. However, the proposed FRLTC algorithm further improves this metric by achieving the lowest mean path loss while maintaining a distribution comparable to ROATC The data in this table clearly show that the FRLTC algorithm always yields the smallest PL compared with the other four algorithms.

**Table 2:** Comparison of path loss of MaxPower, LTRT, TFACR, ROATC and FRLTC algorithms in case of desired degrees of 3 and 4.

Desired degree	Algorithm	Max (dB)	Min (dB)	Average (dB)	Median (dB)
3	MaxPower	88.0	75.5	84.3	85.1
	LTRT	87.9	75.5	83.5	84.6
	TFACR	87.9	75.5	82.6	82.9
	ROATC	86.5	75.5	81.7	81.9
	FRLTC	87.8	75.4	81.5	81.5
4	MaxPower	88.0	75.5	84.3	85.1
	LTRT	87.9	75.5	84.2	85.1
	TFACR	87.9	75.5	83.0	83.9
	ROATC	87.7	75.5	82.8	83.4
	FRLTC	87.8	75.4	82.7	83.3

The influence of the number of sensor nodes on the path loss was also investigated. The results are shown in Fig 13, where we plot the average path loss as a function of the total number of sensor nodes which ranges from 40 to 80. The desired degree for these simulation scenarios was set to 3. The plots in Fig 13 show that the average path loss is almost the same for the case of MaxPower when the number of sensor nodes varies from 40 to 80, the average value of path loss is about 84.3 dB. This is because, in this case, the sensor nodes always operate at maximum transmit power, resulting in poor long links, regardless of whether the sensor node density is sparse or dense or not. When topology control algorithms are used, the PL is significantly reduced, thereby improving the transmission quality in the network. Among the four topology control algorithms used, LTRT, TFACR, ROATC, and FRLTC, the FRLTC algorithm always yielded the smallest path loss with an average value of only 80.5 to 82 dB. The ROATC algorithm consistently achieves the second lowest path loss, outperforming TFACR across all network sizes. However, FRLTC further reduced the average path loss by approximately 0.3–0.5 dB compared with ROATC, indicating a more effective topology-optimization capability. The results are identical for the case in which the desired degree is four, as shown in Fig 14. The FRLTC algorithm exhibited the lowest path loss. These results clearly show that the FRLTC algorithm improves the transmission quality compared with other algorithms.

#### Analyzing network throughput.

In addition to the performance metrics commonly used to evaluate the effectiveness of a topology control algorithm, namely node degree, energy consumption, and path loss, which have been thoroughly investigated in the previous sections, this section further extends the evaluation by analyzing network throughput. This metric is of critical importance because it reflects the data transmission capability of the SDWSN in the IoT, directly indicating the quality of connectivity maintenance and efficiency of network resource utilization under the influence of the proposed topology-control algorithm.

To enable data transmission across the network, the topology control algorithm must operate in conjunction with the routing protocols. In this study, two representative routing protocols, AODV [19] and DSDV [20], were employed, corresponding to the reactive and proactive routing protocols, respectively.

**Fig 15:**
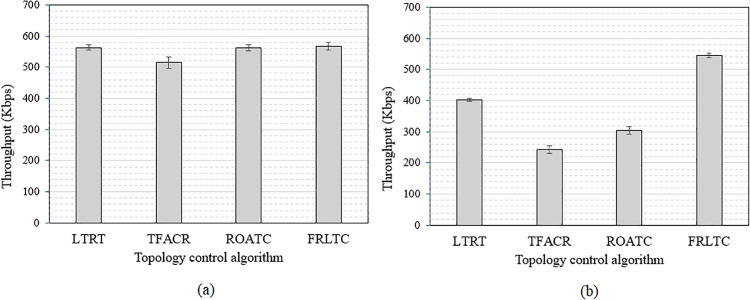
Throughput comparison of LTRL, TFACR, ROATC and FRLTC algorithms in a 50-nodes network under (a) AODV and (b) DSDV routing protocols. Error bars denote the standard deviation over 10 independent simulation runs.

**Fig 16:**
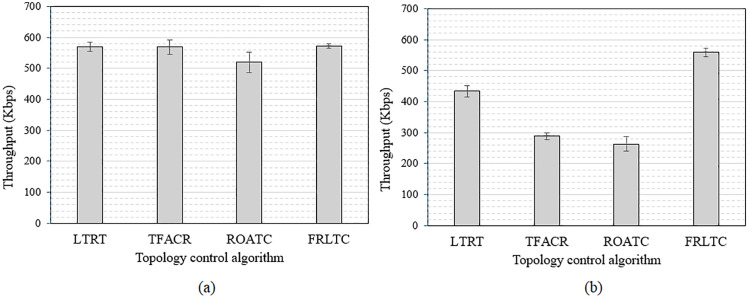
Throughput comparison of LTRL, TFACR, ROATC and FRLTC algorithms in a 60-nodes network under (a) AODV and (b) DSDV routing protocols. Error bars denote the standard deviation over 10 independent simulation runs.

From Figs 15 and 16, it can be observed that network throughput is significantly influenced by both the topology control algorithm and the routing protocol employed. In the 50-node scenario (Fig 15), under AODV routing, FRLTC achieves the highest throughput (567.06 Kbps), followed by ROATC (562.70 Kbps), LTRT (563.78 Kbps), and TFACR (514.46 Kbps). The standard deviations over 10 independent simulation runs were 12.98, 10.23, 8.16, and 17.92 Kbps for FRLTC, ROATC, LTRT, and TFACR, respectively, respectively. This indicates that FRLTC maintains a more efficient network topology, thereby enhancing the end-to-end data transmission capability when combined with AODV’s reactive routing mechanism. Although the throughput of ROATC is very close to that of FRLTC, the proposed algorithm still achieves the highest average throughput. When DSDV was applied, the performance gap became more pronounced: FRLTC still attained the highest throughput (546.02 Kbps), whereas LTRT, ROATC, and TFACR achieved 402.86, 303.70, and 242.70 Kbps, respectively, respectively. The standard deviations over 10 independent simulation runs were 7.42, 5.34, 11.92, and 12.80 Kbps for FRLTC, LTRT, ROATC, and TFACR, respectively. Although the ROATC outperformed the TFACR, it remained considerably below the FRLTC. The substantial degradation of the TFACR under DSDV suggests that this algorithm is less suitable for proactive routing environments, where frequent routing table updates require a highly stable network structure.

A similar trend was observed in the 60-node scenario (Fig 16), although the overall throughput values vary due to increased network density. Under AODV, FRLTC continues to outperform the other algorithms (571.39 Kbps), slightly exceeding TFACR (568.42 Kbps), LTRT (569.87 Kbps), and ROATC (519.00 Kbps). The standard deviations over 10 independent simulation runs were 7.67, 23.85, 14.50, and 33.70 Kbps for FRLTC, TFACR, LTRT, and ROATC, respectively. Notably, the performance gap among the four algorithms becomes narrower compared to the 50-node case, indicating that AODV adapts effectively to topology changes in denser networks, thereby mitigating performance disparities among topology control schemes. However, ROATC exhibited the largest standard deviation, indicating lower throughput stability than the other algorithms. In contrast, under DSDV, FRLTC consistently maintained a superior performance (558.82 Kbps), whereas LTRT (433.31 Kbps), ROATC (262.86 Kbps), and TFACR (288.78 Kbps) exhibited considerably lower throughput The standard deviations over 10 independent simulation runs were 14.42, 18.17, 23.06, and 11.66 Kbps for FRLTC, LTRT, ROATC, and TFACR, respectively. Compared with the ROATC, the FRLTC achieves a significantly higher throughput and exhibits a considerably lower standard deviation, demonstrating its superior stability under proactive routing.

To further assess the reliability of the throughput results, Table 3 summarizes the average throughput and corresponding 95% confidence intervals obtained from 10 independent simulation runs. It can be observed that the confidence intervals of all algorithms are relatively narrow, indicating good repeatability and statistical stability of the simulation results. Under both the AODV and DSDV routing protocols, the proposed FRLTC algorithm consistently achieves the highest average throughput. Moreover, although ROATC exhibits competitive performance under the AODV protocol in the 50-node scenario, its confidence interval is wider than that of FRLTC, suggesting greater performance variability across different simulation runs. In the 60-node scenario, ROATC presents the largest confidence interval among all topology control algorithms under both routing protocols, whereas FRLTC maintains both the highest throughput and a relatively narrow confidence interval. These results further confirm that the proposed FRLTC algorithm not only improves the average network throughput but also provides a more stable performance under different network densities and routing protocols than the other algorithms.

**Table 3:** Average throughput (Kbps) with 95% confidence interval over 10 independent simulation runs.

Network size	Routing	Topology control			
		LTRT	TFACR	ROATC	FRLTC
50 nodes	AODV	563.28±5.84	514.46±12.82	562.70±7.32	567.06±9.29
	DSDV	402.86±3.82	242.70±9.16	303.70±8.53	546.02±5.31
60 nodes	AODV	569.87±10.37	568.42±17.06	519.38±24.10	571.39±5.49
	DSDV	433.31±13.00	288.78±8.34	262.86±16.50	558.82±10.31

Overall, FRLTC demonstrated the best throughput performance for both network scales and routing protocols. AODV generally provides a higher and more stable throughput than DSDV for the LTRT and TFACR, whereas FRLTC is highly compatible with both reactive and proactive routing strategies. These results confirm that the topology optimization mechanism of the FRLTC enhances connectivity quality, reduces transmission conflicts, and preserves link stability, thereby improving the overall network data delivery capacity.

#### Analyzing end-to-end delay.

In addition to throughput, which reflects the overall data transmission capacity of the network, the end-to-end delay (EED) is another critical performance metric, particularly in WSN-IoT systems that require high timeliness and reliability. In many applications, such as real-time monitoring, event detection, and intelligent control, low and stable delays play a decisive role in ensuring the Quality of Service (QoS) of the system. Topology control algorithms not only influence resource utilization and connectivity maintenance, but also directly affect the number of hops, congestion levels, and retransmissions along the routing path, thereby affecting the EED. Therefore, analyzing the end-to-end delay provides a complementary and more comprehensive perspective on the performances of the LTRT, TFACR, ROATC, and FRLTC algorithms under different network scenarios.

**Fig 17:**
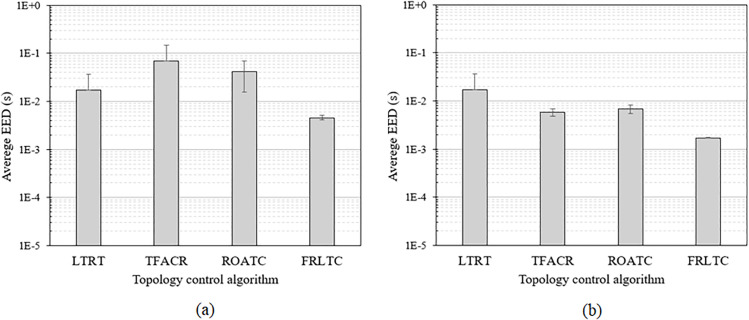
End-to-end delay comparison of LTRL, TFACR, ROATC and FRLTC algorithms in a 50-nodes network under (a) AODV and (b) DSDV routing protocols.

**Fig 18:**
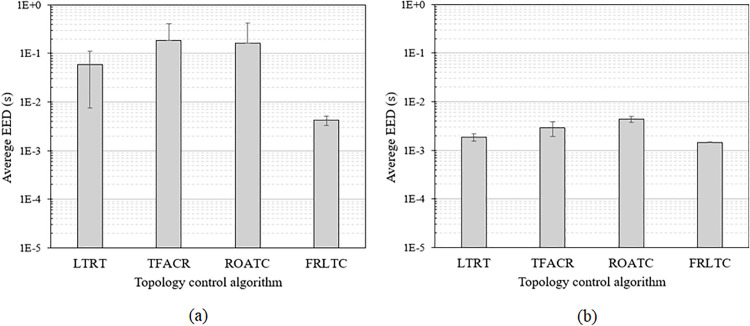
End-to-end delay comparison of LTRL, TFACR, ROATC and FRLTC algorithms in a 60-nodes network under (a) AODV and (b) DSDV routing protocols.

From Figs 17 and 18, it can be observed that the EED varies significantly across topology control algorithms and routing protocols in both the 50-node and 60-node scenarios. In the 50-node network (Fig 17), under AODV, FRLTC achieves the lowest delay, followed by LTRT, ROATC, while TFACR exhibits the highest delay. The standard deviations over 10 independent simulation runs were 0.020, 0.077, 0.027, and 0.001 s for the LTRT, TFACR, ROATC, and FRLTC, respectively. This indicates that the topology constructed by the FRLTC effectively reduces the number of hops, mitigates congestion, and limits the number of packet retransmissions along the routing paths. When DSDV is employed, a similar trend is maintained, although the performance gap among the algorithms becomes slightly smaller, reflecting the relative stability of proactive routing once the routing table is fully established. The standard deviations were 0.02, 0.001, 0.002, and 0.00002 s for the LTRT, TFACR, ROATC, and FRLTC, respectively, indicating that the FRLTC consistently provided the most stable delay performance.

In the 60-node network (Fig 18), as node density increases, the average delay of all algorithms changes due to the higher number of links and increased channel contention. Under AODV, FRLTC maintained the lowest delay, whereas TFACR recorded the highest values, followed closely by ROATC. The standard deviations over 10 independent simulation runs were 0.05, 0.22, 0.26, and 0.001 s for LTRT, TFACR, ROATC, and FRLTC, respectively, demonstrating that FRLTC provides more efficient path optimization and connectivity control in denser network environments. With DSDV, although the overall delay is reduced in certain cases owing to pre-established routing information, FRLTC continues to achieve the lowest average delay with the smallest standard deviation (0.00 s), while the standard deviations of LTRT, TFACR, and ROATC are 3.29E-04, 9.62E-04, 6.28E-04, and 2.04E-05, respectively. The FRLTC outperformed the other algorithms in terms of stability and transmission efficiency. Overall, the EED analysis further confirms that FRLTC not only improves throughput but also ensures faster and more stable data delivery compared to LTRT, ROATC, and TFACR under different network conditions.

**Fig 19:**
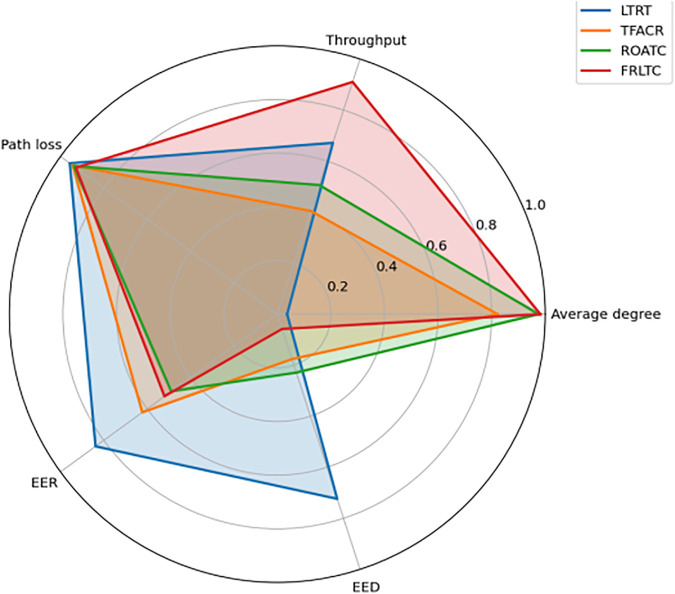
Comparison of the overall performance of topology control algorithms with a simulation scenario of 50 sensor nodes.

**Fig 20:**
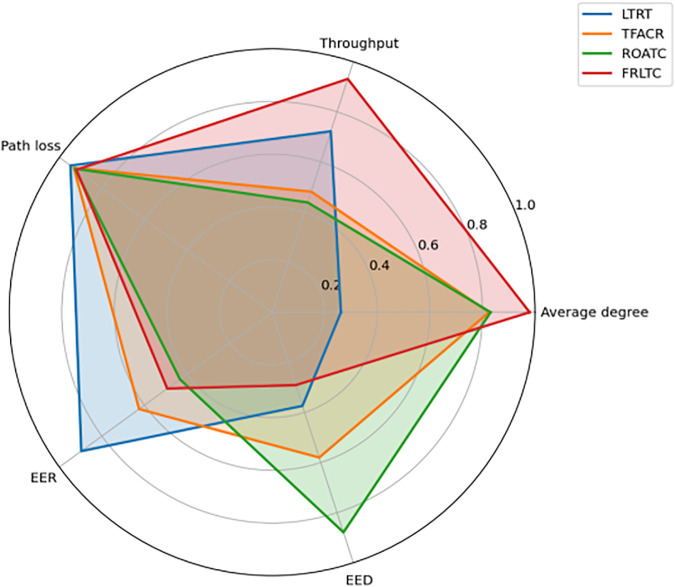
Comparison of the overall performance of topology control algorithms with a simulation scenario of 60 sensor nodes.

Finally, we summarized the performance results of the LRTR, TFACR, ROATC, and FRLTC algorithms, as well as the case without topological control (MaxPower), using a radar plot, as shown in Figs 19 and 20. To construct these graphs, we normalized all metrics node degree, EER, path loss, throughput, and EED to the interval [0, 1]. The EER is expressed as a percentage; therefore, the value is also equivalent to [0, 1]. The path loss was normalized as the ratio of the average path loss to the maximum average path loss. The maximum average path loss is determined by the average path loss when the sensor nodes operate at the maximum transmission power, according to the communication standard used. The throughput is normalized as the ratio of the transmitted traffic to the successfully received traffic. The EED was normalized as the ratio of the actual EED to the maximum EED observed in the network.

The average node degree was normalized as follows:


$$\begin{array}{c} {\overset{―}{k}}_{normalized}=1-\frac{\left|\overset{―}{k}-k_{desired}\right|}{{\overset{―}{k}}_{max}-k_{desired}} \end{array}$$ (9)

where ${\overset{―}{k}}_{max}$ represents the average node degree when the sensor nodes operate at maximum transmit power and $k_{desired}$ represents the desired node degree. For the normalization using (9), when the topology is not controlled, $\overset{―}{k}$ equals ${\overset{―}{k}}_{max}$, ${\overset{―}{k}}_{normalized}$ is equal to 0. In the ideal case of topology control, $\overset{―}{k}$ equals $k_{desired}$, and ${\overset{―}{k}}_{normalized}$ is equal to one.

The results are shown in Figs 19 and 20 indicate that the FRLTC algorithm demonstrates the most balanced and outstanding overall performance, achieving high values in key metrics such as throughput and average degree, while maintaining path loss and end-to-end delay (EED) at reasonable levels. In contrast, LTRT has advantages in terms of the energy efficiency ratio (EER) and lower path loss, but exhibits limitations in terms of the average degree and the delay performance. The TFACR provides competitive performance in terms of path loss and EER; however, its throughput remains significantly lower than that of the FRLTC. The newly introduced ROATC algorithm achieves better overall performance than TFACR, particularly in terms of the average degree, throughput, and path loss. Moreover, ROATC exhibits a lower EED than TFACR in the 50-node scenario and maintains competitive delay performance in the 60-node scenario. Nevertheless, FRLTC consistently outperforms ROATC by simultaneously achieving the highest throughput and average degree, while maintaining the lowest path loss and EED.

As the number of nodes increased from 50 to 60, the shapes of the radar plots changed; however, the overall trend remained consistent, with FRLTC achieving a higher and more stable overall performance, demonstrating better adaptability to increased network density. Although ROATC remains the second-best topology control algorithm in most metrics, its throughput and delay performances degrade more noticeably than those of FRLTC as the network becomes denser. Overall, the two figures clearly illustrate that the FRLTC achieves a well-balanced trade-off among the performance metrics, thereby delivering a more comprehensive network performance compared with the LTRT, TFACR, and ROATC.

## Conclusion

One of the most critical variables determining the performance of an IoT software-defined wireless sensor network (SDWSN) is its topology. This study introduces a novel topology control algorithm for SDWSN in the Internet of Things that uses federated reinforcement learning, namely FRLTC. Our approach depicts the SDWSN as a federated learning system, with each sensor node running a reinforcement learning model to adjust its communication range until the node degree approaches the target value. Another reinforcement learning model is used at the SDN controller to assign the sensor node to learn, bringing the overall network average degree closer to the target degree. Simulation results show that the proposed method outperforms well-known topology control methods in terms of the desired node degree, energy consumption and quality of transmission. This is a meaningful result for improving the SDWSN performance in the IoT.

In future work, we will continue to develop the algorithm by applying deep learning models to improve its performance further. In addition, we consider applying this algorithm to other new-generation wireless network systems in the future.
